# Blood Vessel Occlusion by the Layperson Audiovisual Assist Tourniquet (LAVA TQ) Compared to the Combat Application Tourniquet: Randomized Controlled Trial

**DOI:** 10.5811/westjem.59147

**Published:** 2023-05-09

**Authors:** Craig Goolsby, Nicole Dacuyan-Faucher, Keke Schuler, Annie Lee, Amit Shah, Jeremy Cannon, Curt Kothera

**Affiliations:** # Harbor-UCLA Medical Center, Department of Emergency Medicine, Torrance, California; * David Geffen School of Medicine at UCLA, Los Angeles, California; † The Henry M Jackson Foundation for the Advancement of Military Medicine, Bethesda, Maryland; ‡ Uniformed Services University of the Health Sciences, Bethesda, Maryland; § InnoVital Systems, Calverton, Maryland; ¶ The Lundquist Institute, Torrance, CA; || University of Pennsylvania, Department of Surgery, Philadelphia, Pennsylvania

## Abstract

**Introduction:**

While windlass-rod style tourniquets stop bleeding in limbs when used by skilled responders, they are less successful in the hands of the untrained or not recently trained public. To improve usability, an academic-industry partnership developed the Layperson Audiovisual Assist Tourniquet (LAVA TQ). The LAVA TQ is novel in design and technology and addresses known challenges in public tourniquet application. A previously published multisite, randomized controlled trial of 147 participants showed that the LAVA TQ is much easier for the lay public to use compared to the Combat Application Tourniquet (CAT). This study evaluates the LAVA TQ’s ability to occlude blood flow in humans compared to the CAT.

**Methods:**

This study was a prospective, blinded, randomized controlled trial to demonstrate the non-inferiority of the LAVA TQ to occlude blood flow when applied by expert users compared to the CAT. The study team enrolled participants in Bethesda, Maryland, in 2022. The primary outcome was the proportion of blood flow occlusion by each tourniquet. The secondary outcome was surface application pressure for each device.

**Results:**

The LAVA TQ and CAT occluded blood flow in all limbs (21 LAVA TQ, 100%; 21 CAT, 100%). The LAVA TQ was applied at a mean pressure of 366 millimeters of mercury (mm Hg) (SD 20 mm Hg), and the CAT at a mean pressure of 386 mm Hg (SD 63 mm Hg) (P = 0.14).

**Conclusion:**

The novel LAVA TQ is non-inferior to the traditional windlass-rod CAT in occluding blood flow in human legs. The application pressure of LAVA TQ is similar to that used in the CAT. The findings of this study, coupled with LAVA TQ’s demonstrated superior usability, make the LAVA TQ an acceptable alternative limb tourniquet.

## INTRODUCTION

Immediate hemorrhage control with a limb tourniquet has been life-saving both on and off the battlefield.^1^,^2^ The US military estimates that tourniquets saved more than 1,000 lives in Iraq and Afghanistan, and tourniquets are now being used throughout communities and hospitals across the United States.^1^ The Stop the Bleed (STB) campaign, launched at the White House in 2015, brings these battlefield medical lessons home by teaching the public to control hemorrhage prior to the arrival of an ambulance.^3^,^4^ Rapid tourniquet application—a core STB principle—is now the first-line treatment for extremity hemorrhage in mainstream, public education guidelines.^5^

While the Combat Application Tourniquet (CAT) and other windlass-rod tourniquets can stop bleeding, the public is just 20% successful at applying them without training.^6^ Even with training, a layperson’s ability to apply tourniquets successfully drops to about 50% mere months after training.^7^ While widely available windlass-rod style tourniquets, such as the CAT, can stop bleeding when used by skilled responders, they are not intuitively designed. The non-optimized design makes them difficult for the minimally or untrained public to achieve successful extremity hemorrhage control. Multiple studies have shown poor performance by untrained layperson users, as well as rapid skill loss after training.^6^–^8^

**Figure 1:**
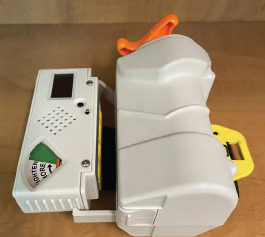
The LAVA TQ^*^: a user-intuitive tourniquet with a seatbelt design and audiovisual instructions. ^*^LAVA TQ, Layperson Audiovisual Assist Tourniquet.

To boost performance by the lay public, a team of academic and industry partners developed the Layperson Audiovisual Assist Tourniquet (LAVA TQ)—the first audiovisual-enabled layperson tourniquet to improve the public’s ability, even if untrained, to save lives from extremity hemorrhage (Figure 1). The design and technology of the LAVA TQ address several known problems with tourniquets applied by the public. The LAVA TQ replaces the sometimes-confusing windlass-rod mechanism found in many standard tourniquets with an intuitive seatbelt design as the tourniquet’s strap and a user-friendly knob for tightening. Like a seatbelt in a vehicle, the LAVA TQ will tighten to snug against the extremity once the belt is clicked into place. This action removes the initial tightening step required in most standard windlass-rod tourniquets, thereby requiring fewer steps to apply. The LAVA TQ guides the user to successful application with a series of lights, color cues, pressure application feedback, and audio instructions.

A recent multisite, international study addressed the *usability* of the LAVA TQ by showing that the untrained public is much more successful at applying the LAVA TQ compared to a CAT (93% vs 22%) on simulated limbs.^9^ In this study we compare the *ability* of the LAVA TQ and CAT to occlude blood flow in human legs. We hypothesized that trained users with medical backgrounds would occlude blood flow during all applications of both the LAVA TQ and CAT on human limbs.

## METHODS

This study was a prospective, blinded, randomized controlled trial to assess non-inferiority of the novel LAVA TQ (experimental arm) to occlude blood flow in human volunteers when compared to the CAT (control arm). The Uniformed Services University (USU) Institutional Review Board (IRB) reviewed and approved this study (USUHS.2020-060), and it is registered on the National Library of Medicine’s Clinical Trials website (clinicaltrials.gov, NCT05504733).

**Population Health Research Capsule**

What do we already know about this issue?

*A layperson is not as successful in applying the Combat Application Tourniquet (CAT) as a skilled responder*.

What was the research question?

*Is the new Layperson Audiovisual Tourniquet (LAVA TQ) non-inferior to CAT in occluding blood flow in human volunteers?*

What was the major finding of the study?

*The LAVA TQ and CAT occluded blood flow in all limbs (n=21 LAVA TQ, 100%; n = 21 CAT, 100%)*.

How does this improve population health?

*Stop the Bleed advocates for public access to trauma supplies. The easier-to-use LAVA TQ, which occludes blood flow comparably to a CAT, might enhance bystander response*.

We performed an a priori power calculation for a parallel group non-inferiority trial. We assumed that all tourniquet applications, in both the control (CAT) and experimental (LAVA TQ) study arms, would occlude blood flow. We then calculated a minimum sample size of 13 applications with each device (26 total) to have 80% power to detect a 10% difference (non-inferiority limit) in performance between the two devices. With IRB permission, we enrolled a total of 21 participants to undergo 42 applications (one device on each leg) by medical professionals. The primary outcome was the proportion of CAT applications compared to the LAVA TQ applications that occluded blood flow in human volunteers. The secondary outcome was the surface pressure of an applied CAT tourniquet compared to an applied LAVA TQ tourniquet in human volunteers.

We recruited healthy participants to undergo application of tourniquets to both of their legs. The exclusion criteria were as follows: age <18 or >65 years old; hypertension; prior vascular surgery; peripheral vascular disease; diabetes; prior lower extremity surgery; active lower extremity infection; any hypercoagulable condition; pregnancy; or any condition in which participants felt they could suffer harm from brief tourniquet application. Participants were recruited via email messages sent to members of the USU community, as well as word of mouth. Participants were not compensated. Studies involving military members and students at USU are reviewed and approved by a series of offices, including the Office of Student Affairs and military chain of command to prevent coercion of participants. For this study protocol we obtained all the routine approvals prior to execution.

**Figure 2:**
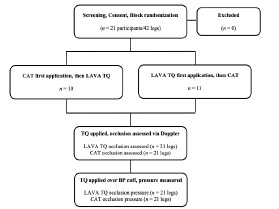
Study flow. *LAVA TQ*, Layperson Audiovisual Assist Tourniquet; *CAT*, Combat Application Tourniquet.

The study team collected all data on May 9, 2022, on the USU campus in Bethesda, Maryland. Twenty-one participants arrived at a pre-scheduled time and then completed a screening questionnaire to verify eligibility (Table 1, Figure 2). After completing screening, each participant reviewed and signed an informed consent document that described the study, potential risks, and their ability to withdraw voluntarily at any time.

The study team randomized each participant and assigned them a participant number. The number was not linked to the participant, and no personally identifying data was collected as part of the study. The randomization occurred in blocks with the first 10 participants assigned to begin the study with CAT application, and the next 11 participants assigned to begin the study with the LAVA TQ application. Study observers evaluating blood flow were blinded to the participants’ randomization. Participants completed a pre-study questionnaire consisting of basic demographic information, height, and weight. Then study team members measured and recorded their resting brachial blood pressure and calf circumferences 10 centimeters (cm) distal to the tibial plateau.

Following the enrollment procedures, participants underwent the first part of the study. The participant removed shoes and socks from both feet and removed or moved any clothing distal to their knees. A trained observer then entered the study room and used a handheld Doppler ultrasound to detect the participant’s dorsalis pedis pulse in each foot. The observer used a surgical marker to place an “X” at the location of the detected pulse on the participant’s foot, and then left the room. One of two trained medical professionals, both with military medical experience and a history of numerous CAT applications, applied either a CAT or LAVA TQ, as determined by randomization, to one of the participant’s legs. The medical professionals had been trained to use LAVA TQ prior to the study. Neither medical professional was involved in the development of the LAVA TQ nor did they have any financial or intellectual property interest in the device. The medical professionals did not assist with study design.

**Figure 3:**
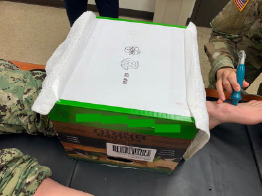
Blinding box used to shield the type of tourniquet from an observer who used handheld Doppler ultrasound to confirm the presence or absence of a dorsalis pedis pulse.

The medical professionals applied the tourniquet at a standardized location on all participants: 10 cm distal to the tibial plateau. They applied the tourniquet until they thought it was tight enough to stop blood flow. As soon as the tourniquet was applied, the medical professional covered the tourniquet and the participant’s lower leg with a blinding box and asked the observer to return to the room (Figure 3). The observer placed the Doppler ultrasound at the previously marked location of the participant’s dorsalis pulse and annotated a checklist to indicate if a pulse was present. The observer left the room, and the medical professional then removed the blinding box and tourniquet. The medical professional followed the same procedures to place the other type of tourniquet on the opposite leg.

After performing the experiment to determine the ability of the CAT and LAVA TQ to occlude blood flow, the participants underwent a second application of each device to determine their respective surface application pressures. The medical professional placed a neonatal blood pressure cuff (Neonate #1, single hose), which had been connected to an external gas pressure sensor system (Vernier Gas Pressure sensor, LabQuest 3 interface [Vernier Science Education, Beaverton, OR]), on the participant’s anterior lower leg 10 cm distal to the tibial plateau, with the length of the cuff oriented in the limb circumference direction. This blood pressure cuff was then wrapped lightly with an elastic wrap bandage to hold it in place. Then the external pressure gauge was zeroed, and the medical professional applied either the CAT or LAVA TQ, based on randomization, to the participant’s leg with the attached neonatal blood pressure cuff.

The study team chose a neonatal blood pressure cuff as it fit under the band of either the CAT or LAVA TQ. The medical professional applied the device until they thought it would stop blood and covered the participant’s leg and tourniquet with the same blinding box used previously. The observer then entered the room and used the Doppler ultrasound to confirm the presence or absence of a dorsalis pedis pulse. If the pulse was absent, the medical professional recorded the surface pressure measured by the blood pressure cuff and gauge. If the pulse had been present, the medical professional would tighten and re-check. The observer left the room, and the medical professional removed the box and tourniquet. The medical professional then repeated the same series of steps using the other tourniquet on the participant’s opposite leg.

## RESULTS

The 21 study participants had a mean age of 28 years (range 22–51), a mean weight of 81 kilograms (kg) (range 50–132 kg), and 26% were female (Table 1). For the study’s primary outcome, the medical professionals occluded blood flow in all participants in both the experimental LAVA TQ arm (21, 100%), and the control CAT arm (21, 100%) (Table 2). For the study’s secondary outcome of tourniquet application pressure, the LAVA TQ was applied at a mean pressure of 366 millimeters of mercury (mm Hg) (SD 20 mm Hg), and the CAT was applied at a mean pressure of 386 mm Hg (SD 63 mm Hg) (Table 2). The difference in application pressures was not statistically significant (*P* = 0.14).

## DISCUSSION

The novel LAVA TQ is non-inferior to a traditional windlass-rod CAT in occluding blood flow in human legs. For skilled users, the CAT has proven effective at stopping life-threatening hemorrhage in limbs, and the LAVA TQ is equally effective in occluding blood flow in this experiment in human limbs. While the primary anticipated benefit of the LAVA TQ is enhanced usability compared to the CAT, it is essential to document the device’s performance in occluding blood flow. The use of handheld Doppler ultrasound for measuring blood flow has been validated previously.^10^,^11^ Since all blood flow was occluded in both the experimental and control study arms, there is little reason to suspect bias from the blinded observers. None of the study’s observers, participants, or medical professionals have any financial interest in the LAVA TQ.

Tourniquets are now widely recommended for use by the public as the first-line treatment for life-threatening extremity hemorrhage.^5^,^12^ While existing standard tourniquets can occlude blood flow, multiple studies have shown that laypersons have difficulty applying them if they have never been trained to apply a tourniquet, if they have not been trained within the prior several weeks, or if they do not have adjunctive aids to assist them.^6^,^13^–^14^ Since most people in the US will not receive ongoing or refresher STB training, finding alternative methods or devices to assist tourniquet application at the point of injury is essential. In fact, using audiovisual instructions to assist tourniquet application was one of the five original goals of the Stop the Bleed campaign.^4^ The LAVA TQ is the first device to bring this goal to reality, and this study demonstrates that it can accomplish its essential function of stopping blood flow on a human extremity.

While the difference in pressure applied to the limbs when applying either device was not statistically different in this study, other studies have demonstrated significantly higher application pressures for CAT.^15^ It is important to note that the pressures reported here are not necessarily the minimum pressure required to achieve occlusion (ie, the pressure above which there is an absence of arterial pulse). In this study, medical professionals used their judgment to assess adequate tightness but were not reacting to patient parameters such as ongoing bleeding. It is possible that the measured pressure differences were similar in part because the medical professionals were familiar with the CAT application and applied a similar tightness with the LAVA TQ. It is expected that the greater strap width in the LAVA TQ compared to the CAT will occlude arterial flow at lower application pressures, which might reduce the possibility of nerve injury or other damage compared to traditional windlass-rod designs. Additionally, the LAVA TQ design allows for pressure increase in smaller increments than the CAT, further reducing the risk of over-tightening.

## LIMITATIONS

This study has limitations. These tourniquet applications were performed in a laboratory setting, and findings could vary with actual injury. The study does not assess durability factors or usability for either device. Trained professionals applied the devices in this study, rather than the lay public who may use the LAVA TQ eventually. We used professionals to ensure that we could attribute an inability of either device to occlude blood flow to device design, rather than human application error. The neonatal blood pressure cuff measuring system has been described previously but has limitations.^15^ The use of air in the measurement cuffs, rather than an incompressible liquid, could cause some variation from the actual pressures applied due to cuff deformation or air leakage. We anticipate that any errors due to cuff performance would affect the LAVA TQ and CAT equally, but this is not certain.

Tourniquet application is a painful procedure. We did not survey the participants specifically about application pain of the two tourniquets. However, multiple participants remarked spontaneously to the study team that the LAVA TQ was less painful than the CAT. This is likely due to the wider strap on the LAVA TQ compared to the CAT; this could be a useful area for future investigation.

## CONCLUSION

The novel Layperson Audiovisual Assist Tourniquet is non-inferior to the traditional windlass-rod Combat Application Tourniquet in occluding blood flow in human legs when applied by a trained medical professional. The surface application pressure of LAVA TQ is similar to that of the CAT in healthy volunteer study subjects. The findings of this study confirm that the LAVA TQ is an acceptable alternative limb tourniquet for occluding blood flow. Study of the LAVA TQ’s performance in real-world bleeding situations is warranted.

## Figures and Tables

**Table 1:** Participant demographics.

	All (N = 19)^a^

	n (%)
Age (years), M (range)	28 (22–51)
Gender	
Female	5 (26)
Race^b^	
Asian or Pacific Islander	3 (16)
Black	0 (0)
White	18 (95)
Other	1 (5)
Ethnicity	
Hispanic origin	5 (26)
Not of Hispanic origin	14 (74)
Height (cm), M (range)	175 (152–196)
Weight (kg), M (range)	81 (50–132)
Right leg circumference (cm), M (range)	38 (27–47)
Left leg circumference (cm), M (range)	38 (27–47)

a Two participants did not complete the demographic information.

b Mixed race participants were counted for multiple races.

*cm*, centimeter; *kg*, kilogram.

**Table 2:** Blood flow occlusion in the Layperson Audiovisual Assist Tourniquet vs the Combat Appllcation Tourniquet.

	LAVA TQ (n = 21) n (%)	CAT (n = 21) n (%)	P-value
Successful occlusion	21 (100)	21 (100)	
Occlusion pressure (mm Hg), Mean (SD)	366 (20)	386 (63)	0.14

*LAVA TQ*, Layperson Audiovisual Assist Tourniquet; *CAT*, Combat Application Tourniquet; *mm Hg*, millimeters of mercury.

## References

[1] Blackbourne LH, Baer DG, Eastridge BJ (2012). Military medical revolution: prehospital combat casualty care. J Trauma Acute Care Surg.

[2] Scerbo MH, Holcomb JB, Taub E (2017). The trauma center is too late: Major limb trauma without a pre-hospital tourniquet has increased death from hemorrhagic shock. J Trauma Acute Care Surg.

[3] Rasmussen TE, Baer DG, Goolsby C (2016). The giving back: battlefield lesson to national preparedness. J Trauma Acute Care Surg.

[4] JEMS Staff What the White House’s Stop the Bleed campaign means for EMS.

[5] Pellegrino JL, Charlton NP, Carlson JN (2020). 2020 American Heart Association and American Red Cross Focused Update for First Aid. Circulation.

[6] Goolsby C, Branting A, Chen E (2015). Just-in-time to save lives: a pilot study of layperson tourniquet application. Acad Emerg Med.

[7] Goralnick E, Chaudhary MA, McCarty JC (2018). Effectiveness of instructional interventions for hemorrhage control readiness for laypersons in the Public Access and Tourniquet Training Study (PATTS): a randomized clinical trial. JAMA Surgery.

[8] Ross EM, Mapp JG, Redman TT (2018). The tourniquet gap: a pilot study of the intuitive placement of three tourniquet types by laypersons. J Emerg Med.

[9] Goolsby C, Jonson CO, Goralnick E (2022). The untrained public’s ability to apply the Layperson Audiovisual Assist Tourniquet vs a Combat Application Tourniquet: a randomized controlled trial. J Am Coll Surg.

[10] Laurentino GC, Loenneke JP, Mouser JG (2020). Validity of the handheld Doppler to determine lower-limb blood flow restriction pressure for exercise protocols. J Strength Cond Res.

[11] Swan KG, Wright DS, Barbagiovanni SS (2009). Tourniquets revisited. J Trauma.

[12] Singletary EM, Zideman DA, Bendall JC (2020). 2020 International Consensus on First Aid Science with Treatment Recommendations. Resuscitation.

[13] Goolsby C, Chen E, Branting A (2016). Analysis of layperson tourniquet application using a novel color-coded device. Disaster Med Public Health Prep.

[14] Goolsby C, Rojas LE, Rodzik RH (2020). High-school students can stop the bleed: a randomized, controlled educational trial. Acad Pediatr.

[15] Wall PL, Duevel DC, Hassan MB (2013). Tourniquets and occlusion: the pressure of design. Mil Med.

